# Constructing patch-based ligand-binding pocket database for predicting function of proteins

**DOI:** 10.1186/1471-2105-13-S2-S7

**Published:** 2012-03-13

**Authors:** Lee Sael, Daisuke Kihara

**Affiliations:** 1 Department of Biological Sciences, Purdue University, West Lafayette, IN, 47907, USA; 2 Department of Computer Science, Purdue University, West Lafayette, IN, 47907, USA

## Abstract

**Background:**

Many of solved tertiary structures of unknown functions do not have global sequence and structural similarities to proteins of known function. Often functional clues of unknown proteins can be obtained by predicting small ligand molecules that bind to the proteins.

**Methods:**

In our previous work, we have developed an alignment free local surface-based pocket comparison method, named Patch-Surfer, which predicts ligand molecules that are likely to bind to a protein of interest. Given a query pocket in a protein, Patch-Surfer searches a database of known pockets and finds similar ones to the query. Here, we have extended the database of ligand binding pockets for Patch-Surfer to cover diverse types of binding ligands.

**Results and conclusion:**

We selected 9393 representative pockets with 2707 different ligand types from the Protein Data Bank. We tested Patch-Surfer on the extended pocket database to predict binding ligand of 75 non-homologous proteins that bind one of seven different ligands. Patch-Surfer achieved the average enrichment factor at 0.1 percent of over 20.0. The results did not depend on the sequence similarity of the query protein to proteins in the database, indicating that Patch-Surfer can identify correct pockets even in the absence of known homologous structures in the database.

## Background

An increasing number of protein structures of uncharacterized proteins have been solved by structural genomics projects. As of June, 2011, there are 3321 structures of unknown function in the Protein Data Bank (PDB). Elucidating function of these proteins is an importation task for bioinformatics. To predict protein function from structure, we have recently developed an alignment free local pocket surface comparison method for predicting the type of ligand that is likely to bind to a query protein [1]. The algorithm, named Patch-Surfer, represents a binding pocket as a combination of segmented surface patches, each of which is characterized by its shape, the electrostatic potential, the hydrophobicity, and the concaveness. A query pocket, represented as a group of patches, is compared with a database of pockets of known binding ligand molecules, and binding ligand prediction is made by summarizing similar pockets retrieved from the database. Representing a pocket by a set of patches was shown to be effective in tolerating difference in global pocket shape while capturing local similarity of pockets. The shape and the physicochemical property of surface patches are represented using the 3D Zernike descriptor (3DZD), a series expansion of mathematical 3D function. In this work, we constructed a large database of ligand binding pockets, which contains a diverse set of pockets. We evaluated the performance of Patch-Surfer on the database in terms of the enrichment factor of correct ligand binding pockets retrieved from the database for query pockets.

## Methods

### The Patch-Surfer method for binding ligand prediction

Here we briefly describe Patch-Surfer algorithm. Please refer to the original paper for more details [1]. Given a query protein structure, the surface is computed and a pocket region is extracted. If the binding pocket of the protein is not known, we can predict it using a protein pocket detection algorithm, such as Visgrid [2]. The pocket is segmented to surface patches where four features of each patch, geometrical shape, the surface electrostatic potential, the hydrophobicity, and the concaveness [2], are encoded with 3DZD for efficient storage and comparison. Thus, a pocket is represented by a set of surface patches [1,3]. The 3DZD is a series expansion of a 3D function, which allows compact and rotationally invariant representation of a 3D object (*i.e*. a 3D function) [4]. To compute the 3DZD for a patch, a patch is mapped on a 3D grid and grid points that overlap with the patch are marked with either 1 (for indicating the geometrical shape) or physicochemical values to represent. The assigned values in the 3D grid are considered as a 3D function, *f(**x**)*, which is expanded into a series in terms of Zernike-Canterakis basis defined as follows:

(1) $$Z_{nl}^{m}\left(r,\vartheta ,\phi \right)=R_{nl}\left(r\right)Y_{l}^{m}\left(\vartheta ,\phi \right)$$

where -*l < m <l*, 0 ≤ *l *≤ *n*, and (*n*-*l*) even. $Y_{l}^{m}\left(\vartheta ,\phi \right)$ are the spherical harmonics and *R_nl_*(*r*) is the radial function constructed in a way that $Z_{nl}^{m}\left(r,\vartheta ,\phi \right)$ can be converted to polynomials in the Cartesian coordinates, $Z_{nl}^{m}\left(x\right)$. To obtain the 3DZD of *f(**x**)*, first 3D Zernike moments are computed:

(2) $$\Omega_{nl}^{m}=\frac{3}{4\pi }\int_{\left|x\right|\leq 1}f\left(x\right){\bar{Z}}_{nl}^{m}\left(x\right)dx$$

Then, the 3DZD, *F_nl_*, is computed as norms of vectors Ω*_nl_*. The norm gives rotational invariance to the descriptor:

(3) $$F_{nl}^{}=\sqrt{\overset{m=l}{\underset{m=-l}{\sum }}{\left(\Omega_{nl}^{m}\right)}^{2}}$$

*n *defines the range of *l *and a 3DZD is a series of invariants (Eqn. 3) for each pair of *n *and *l*, where *n *ranges from 0 to the specified order. We use order *n *= 15 (72 invariants) in the local surface patch comparison. The shape and the concaveness are represented by a vector of 72 invariant values while vectors for the electrostatic potential and the hydrophobicity have 144 invariants.

Next, the query pocket is compared to known pockets stored in the database. In the database, each pocket is also represented as a set of surface patches. For example, ATP binding pockets are represented with, on average, 29.5 patches. Given the query pocket and a pocket in the database, the pocket comparison process first identifies similar patches between the two pockets using a modified bipartite matching algorithm. Two options were tested for the matching stage: the first approach matches all patches while the other approach matches only patches that are more similar than the predefined distance threshold value. The similarity of the two pockets is measured with linearly combined scoring terms between the matched patches.

### Constructing database of representative ligand binding pockets

Representative pockets are selected as follows. A list of 5,438 non-redundant protein structures complexed with ligand molecules extracted from PDB was obtained from the Protein-Small-Molecule DataBase http://compbio.cs.toronto.edu/psmdb/downloads/CPLX_25_0.85_7HA.list[5]. From this list, first, we removed all ligands that consist of less than 7 heavy atoms. Then, two ligands which bind to the same protein were grouped together if a pair of atoms, one from each ligand, are closer than 4.0 Å. We further filtered out ligands that are closer than 1.4 Å to the protein, because they bind covalently to proteins. Also, ligand molecules that are more distant than 3.5 Å to any of the protein heavy atoms were removed, as they are not physically interacting with the protein. Finally, we obtained 9,393 pockets structures which bind 2707 different types of ligand molecules.

### Obtaining weighting factors for scoring function

The distance between patch A in the query pocket and patch B in a pocket in the database is defined as:

(4) $$pdist\left(A,B\right)=\underset{t\in \left\{shape,hyd,ele,conc\right\}}{\sum }w_{t}^{B}\times L2\left(3dzd\left(A,t\right),3dzd\left(B,t\right)\right),$$

where *L2 *is the L2 norm (the Euclidian distance) between the *3DZD*s of patch *A *and *B *in terms of the surface property *t*, which is either the geometrical shape, hydrophobicity, the surface electrostatic potential, or the concaveness [2] of the patch. *w_t_^B ^*is the weighting factor for the property *t*, which depend on the patch B from the database. These weights for each patch in each ligand molecules were computed using the average (*avg*) and the standard deviation (*std*) of the Euclidian distance of the patches at the equivalent position (i.e. patches whose closest ligand atom are the same) in the same ligand binding pockets in the database. Weight of a patch *P *for surface property *t *∈ {*shape, hyd, ele, con*} is defined as follows:

(5) $$ws_{t}^{P}=\frac{1\;/)\;\left(avg_{t}+2std_{t}\right)}{\underset{a\in \left\{shape,hyd,ele,con\right\}}{\sum }1\;/)\;\left(avg_{a}+2std_{a}\right)}$$

The average and the standard deviation are used to normalize the difference in the distribution of the four properties.

### Test dataset

We tested the performance of Patch-Surfer using a test dataset that consists of 75 protein pockets, each of which binds to one of the following seven ligands: adenosine monophosphate (AMP) (9 pockets), adenosine-5'-triphosphate (ATP) (14 pockets), flavin adenine dinucleotide (FAD) (10 pockets), flavin mononucleotide (FMN) (6 pockets), alpha- or beta-d-glucose (GLC) (5 pockets), heme (HEM) (16 pockets), and nicotinamide adenine dinucleotide (NAD) (15 pockets).

### Enrichment factor

We used the enrichment factor to evaluate how well Patch-Surfer retrieves binding pockets of the same binding ligand for query pockets. The enrichment factor (EF) describes the ratio of correctly retrieved pockets relative to the percentage of the database entries scanned:

(6) $$EF^{x}=\left(N_{P}^{x}\;/)\;N_{x}\right)/)\left(T_{P}\;/)\;T_{DB}\right),$$

where *T_P _*is the total number of pockets that bind the ligand type *P *in the database of the size *T_DB_*, *N^x^_P _*is the number of pocket for the ligand type *P *ranked within the top *x *percent by the database search method (Patch-Surfer) and *N_x _*is the total number of retrieved pockets ranked in the top *x *percent of the database.

## Results and discussion

### Pocket retrieval results

**Figure 1:**
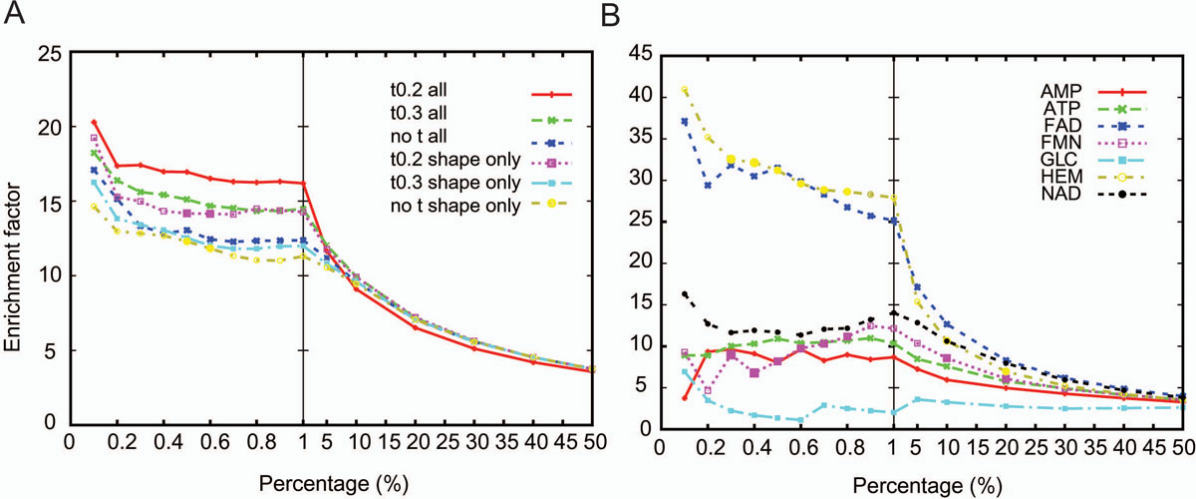
**Enrichment factor calculated for different percentiles**. A, the average EF of 75 query pockets using different combinations of the distance threshold and the surface properties. t0.2, t0.3 shows results using the threshold distance of 0.2 and 0.3, respectively; "no t" shows the result when no threshold is used. Two surface property combinations are used: all four properties, the shape, hydrophobicity, the electrostatic potential, and the visibility, and only using the shape information. B, EF for each of the ligand types in the test dataset using the distance threshold of 0.2 and all the four surface properties.

Patch-Surfer was run with six different settings: using all the four properties or using only the shape information combined with three different distance thresholds for matching patches, 0.2, 0.3, and no threshold for the patch distance (Eqn. 4). Using the threshold value of 0.2, only similar surface patches with the distance closer than 0.2 are matched while the no threshold option matches the maximum number of pairs between two pockets regardless of their distance (i.e. all the patches in the smaller pocket are matched to patches in the larger pocket). The results (Figure 1A) show that first, using all the four properties showed better EF than just using the shape information, and second, using the threshold value of 0.2 performed best among the three choices tested for the distance threshold. The best retrieval was observed when all the patch properties and the threshold of 0.2 were used. Figure 1B shows the EF of each ligand types using Patch-Surfer with the threshold distance of 0.2 and all the four properties. The HEM and the FAD showed very high EF values of over 30 at early ranks. Patch-Surfer performed relatively poorly for GLC. The reason for this is that there are twenty other ligands that are similar to GLC in the database, according to the Tanimoto coefficient (higher than 0.85).

### Effect of the sequence identity to the enrichment factor

**Figure 2:**
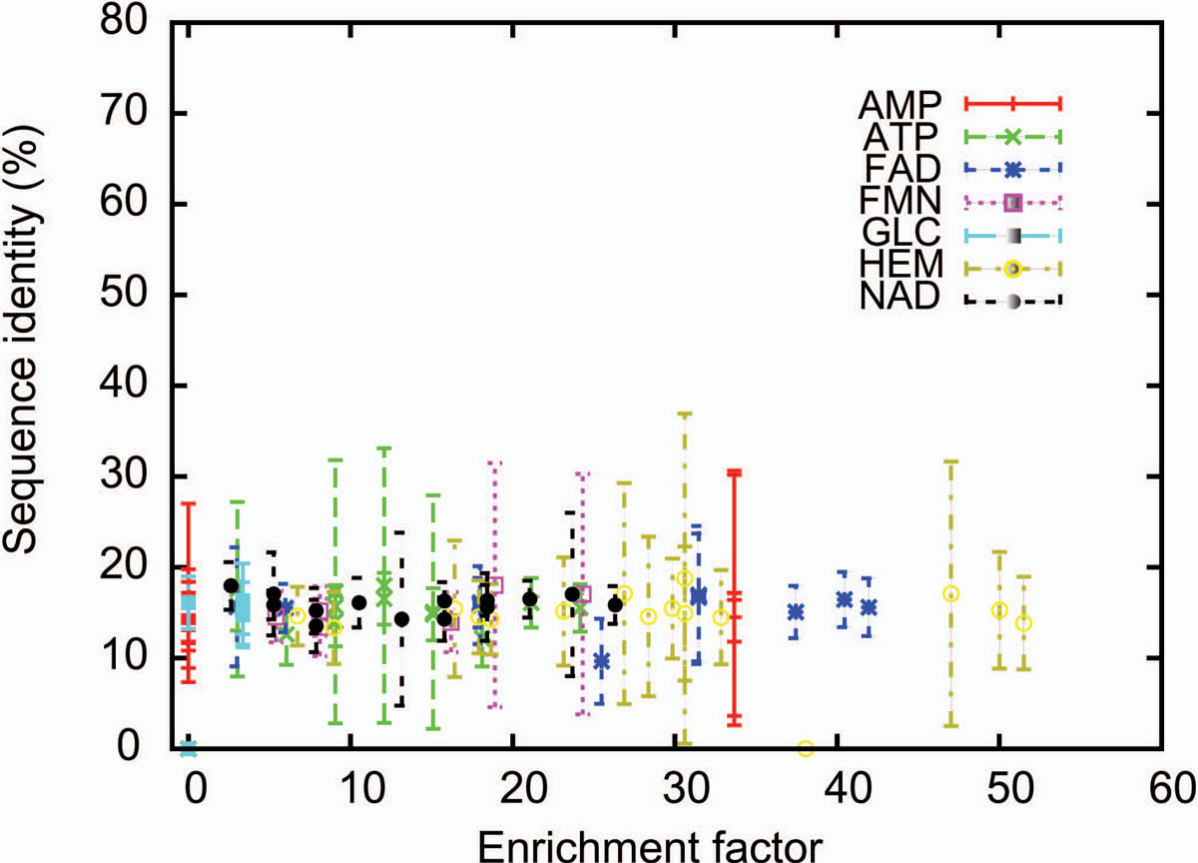
**Effect of the sequence similarity in retrieval performance**. The sequence identity against the enrichment factor is plotted for all 75 test pockets. The dots in the center of bars show the average sequence identity between a query protein and the proteins that bind the same ligand molecule as the query protein. The boundary of the bars shows the standard deviation of the sequence identity for each query protein.

In Figure 2 we show the EF (at 1.0%) of each of the 75 query proteins relative to the sequence identity between the query proteins to the proteins in the database that bind to the same ligand molecules. The correlation coefficient between the average sequence identity to their EF values is 0.05. The plot clearly shows that there is no dependency between the sequence identity and the EF values. Patch-Surfer can retrieve binding pockets of the same ligand type even without having highly similar proteins in the database.

### Binding ligand prediction examples

**Figure 3:**
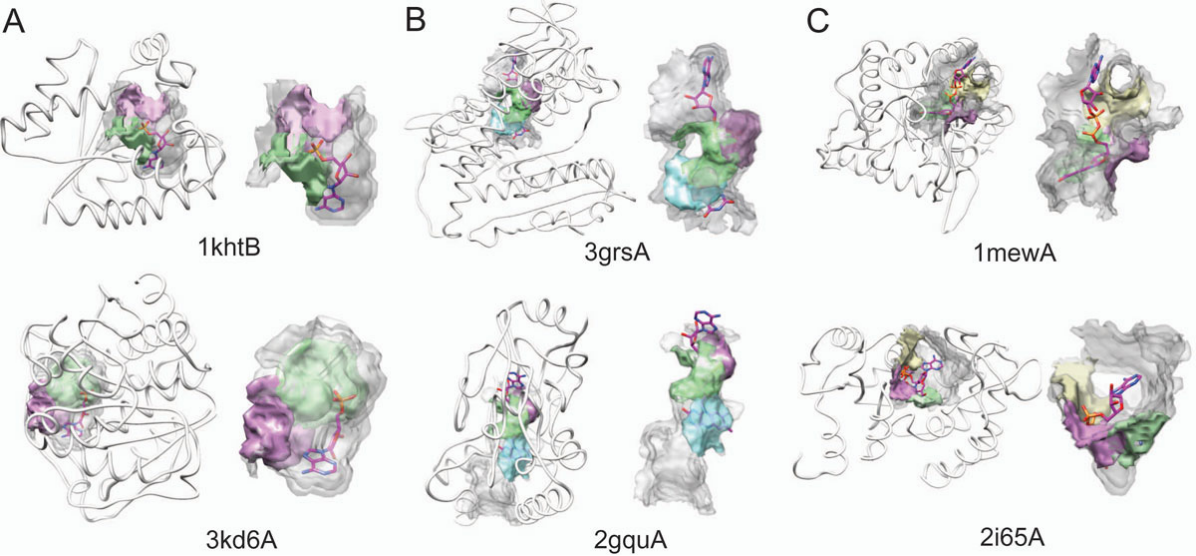
**Examples of retrieved binding pockets**. The proteins shown on the top at each panel are the query protein and the bottom row shows the proteins that were retrieved at the top rank by Patch-Surfer from the database. A, a pair of AMP binding proteins and their binding pockets, 1khtB and 3kd6A. B, FAD binding proteins, 3grsA and 2gqu. C, NAD binding proteins, 1mewA and 2i65A. The color of the patches shows corresponding matched patches from the two pockets.

Figure 3 shows three examples of the query and database protein pairs that were ranked at the 1^st ^in the retrieval by Patch-Surfer. The three pairs bind AMP, FAD, and NAD, respectively. The sequence identity between the AMP binding proteins (Figure 3A) is only15.8%, FAD binding proteins (Figure 3B) has the sequence identity of 16.8%, and NAD binding protein pairs (Figure 3C) has the sequence identity of 15.3%. The pairs of proteins have different overall backbone structure, thus methods that compare global protein structure or the global pocket shape would not capture their similarity.

## Conclusions

We constructed a large database of representative ligand binding pockets for Patch-Surfer. The sufficiently high EF achieved by Patch-Surfer shows that the method is able to retrieve pockets of the same binding ligand from the large database even in absence of homologous proteins in the database. We are currently building a web server for easy access to Patch-Surfer.

## Competing interests

The authors declare that they have no competing interests.

## Authors' contributions

DK conceived the study and SL and DK designed the experiments. SL executed the experiments. SL drafted the manuscript and DK revised it critically. All authors read and approved the manuscript.

## References

[B1] Sael L Kihara D Binding ligand prediction for proteins using partial matching of local surface patches Int J Mol Sci 2010 11 5009 5026 10.3390/ijms11125009 21614188 PMC3100846

[B2] Li B Turuvekere S Agrawal M La D Ramani K Kihara D Characterization of local geometry of protein surfaces with the visibility criterion Proteins 2008 71 670 683 10.1002/prot.21732 17975834

[B3] Sael L Kihara D Characterization and classification of local protein surfaces using self-organizing map International Journal of Knowledge Discovery in Bioinformatics (IJKDB) 2010 1 32 47

[B4] Canterakis N 3D Zernike moments and Zernike affine invariants for 3D image analysis and recognition Proc 11th Scandinavian Conference on Image Analysis 1999 85 93

[B5] Wallach I Lilien R The protein-small-molecule database, a non-redundant structural resource for the analysis of protein-ligand binding Bioinformatics 2009 25 615 620 10.1093/bioinformatics/btp035 19153135

